# Supplementary material for: MtDNA species-level phylogeny and delimitation support significantly underestimated diversity and endemism in the largest Neotropical cichlid genus (Cichlidae: Crenicichla)
Source: PeerJ. 2021 Nov 9;9:e12283. doi: 10.7717/peerj.12283 (PMC8588857; doi:10.7717/peerj.12283)
Supplement: Supplemental Information 1 [file peerj-09-12283-s001.doc]

MtDNA species-level phylogeny and delimitation support significantly underestimated diversity and endemism in the largest Neotropical cichlid genus (Cichlidae: *Crenicichla*)

**Appendices:**

**Appendix 1 Nominal species of *Crenicichla* and *Teleocichla* (in alphabetical order)**

**in Tabular form in: Table S1**

***Crenicichla*, all described species (132):**

**valid: 96 (synonyms: 36)**

**Included in present study (in green): 72**

*acutirostris,* *Crenicichla,* Günther, 1862 (valid)

*adspersa,* *Crenicichla,* Heckel, 1840 (valid)

*alta,* *Crenicichla,* Eigenmann, 1912 (valid)

*anamiri,* *Crenicichla,* Ito & Py-Daniel, 2015 (valid)

*anthurus,* *Crenicichla,* Cope, 1871 (valid)

*brasiliensis marmorata,* *Crenicichla,* Pellegrin, 1904 (valid)

*brasiliensis,* *Perca,* Bloch, 1792 (valid)

*britskii,* *Crenicichla,* Kullander, 1982 (valid)

*cametana,* *Crenicichla,* Steindachner, 1911 (valid)

*celidochilus,* *Crenicichla,* Casciotta, 1987 (valid)

*cincta,* *Crenicichla,* Regan, 1905 (valid)

*compressiceps,* *Crenicichla,* Ploeg, 1986 (valid)

*conibos,* *Cycla,* Castelnau, 1855 (valid)

*coppenamensis,* *Crenicichla,* Ploeg, 1987 (valid)

*cyanonotus,* *Crenicichla,* Cope, 1870 (valid)

*cyclostoma,* *Crenicichla,* Ploeg, 1986 (valid)

*dandara,* *Crenicichla,* Varella & Ito, 2018 (valid)

*empheres,* *Crenicichla,* Lucena, 2007 (valid)

*frenata,* *Crenicichla,* Gill, 1858 (valid)

*gaucho,* *Crenicichla,* Lucena & Kullander, 1992 (valid)

*geayi,* *Crenicichla,* Pellegrin, 1903 (valid)

*gillmorlisi,* *Crenicichla,* Kullander & Lucena, 2013 (valid)

*hadrostigma,* *Crenicichla,* Lucena, 2007 (valid)

*haroldoi,* *Crenicichla,* Luengo & Britski, 1974 (valid)

*heckeli,* *Crenicichla,* Ploeg, 1989 (valid)

*hemera,* *Crenicichla,* Kullander, 1990 (valid)

*hu,* *Crenicichla,* Piálek, Říčan, Casiotta & Almiron, 2010 (valid)

*hummelincki,* *Crenicichla,* Ploeg, 1991 (valid)

*chicha,* *Crenicichla,* Varella, Kullander & Lima, 2012 (valid)

*igara,* *Crenicichla,* Lucena & Kullander, 1992 (valid)

*iguapina,* *Crenicichla,* Kullander & Lucena, 2006 (valid)

*iguassuensis,* *Crenicichla,* Haseman, 1911 (valid)

*inpa,* *Crenicichla,* Ploeg, 1991 (valid)

*isbrueckeri,* *Crenicichla,* Ploeg, 1991 (valid)

*jaguarensis,* *Crenicichla,* Haseman, 1911 (valid)

*jegui,* *Crenicichla,* Ploeg, 1986 (valid)

*johanna* var. B *strigata,* *Crenicichla,* Günther, 1862 (valid)

*johanna,* *Crenicichla,* Heckel, 1840 (valid)

*jupiaensis,* *Crenicichla,* Britski & Luengo, 1968 (valid)

*jurubi,* *Crenicichla,* Lucena & Kullander, 1992 (valid)

*labrina,* *Cychla,* Spix & Agassiz, 1931 (valid)

*lacustris,* *Cycla,* Castelnau, 1855 (valid)

*lenticulata,* *Crenicichla,* Heckel, 1840 (valid)

*lepidota,* *Crenicichla,* Hensel, 1870 (valid)

*lucenai,* *Crenicichla,* Mattos, Schindler, Ottoni & Cheffe, 2014 (valid)

*lucius,* *Crenicichla,* Cope, 1871 (valid)

*lugubris,* *Crenicichla,* Heckel, 1840 (valid)

*macrophthalma,* *Crenicichla,* Heckel, 1840 (valid)

*maculata,* *Crenicichla,* Kullander & Lucena, 2006 (valid)

*mandelburgeri,* *Crenicichla,* Kullander, 2009 (valid)

*minuano,* *Crenicichla,* Lucena & Kullander, 1992 (valid)

*missioneira,* *Crenicichla,* Lucena & Kullander, 1992 (valid)

*monicae,* *Crenicichla,* Kullander & Varella, 2015 (valid)

*mucuryna,* *Crenicichla,* von Ihering, 1914 (valid)

*multispinosa,* *Crenicichla,* Pellegrin, 1903 (valid)

*nickeriensis,* *Crenicichla,* Ploeg, 1987 (valid)

*niederleinii,* *Acharnes,* Holmberg, 1891 (valid)

*notophthalmus,* *Crenicichla,* Regan, 1913 (valid)

*pellegrini,* *Crenicichla,* Ploeg, 1991 (valid)

*percna,* *Crenicichla,* Kullander, 1991 (valid)

*phaiospilus,* *Crenicichla,* Kullander, 1991 (valid)

*ploegi,* *Crenicichla,* Varella, Loeb, Lima & Kullander, 2018 (valid)

*prenda,* *Crenicichla,* Lucena & Kullander, 1992 (valid)

*proteus,* *Crenicichla,* Cope, 1871 (valid)

*punctata,* *Crenicichla,* Hensel, 1870 (valid)

*pydanielae,* *Crenicichla,* Ploeg, 1991 (valid)

*regani,* *Crenicichla,* Ploeg, 1989 (valid)

*reticulatus,* *Batrachops,* Heckel, 1840 (valid)

*rosemariae,* *Crenicichla,* Kullander, 1997 (valid)

*santosi,* *Crenicichla,* Ploeg, 1991 (valid)

*saxatilis semicincta,* *Crenicichla,* Steindachner, 1892 (valid)

*saxatilis* var. *albopunctata,* *Crenicichla,* Pellegrin, 1904 (valid)

*saxatilis,* *Sparus,* Linnaeus, 1758 (valid)

*scottii,* *Batrachops,* Eingenmann, 1907 (valid)

*sedentaria,* *Crenicichla,* Kullander, 1986 (valid)

*semifasciatus,* *Batrachops,* Heckel, 1840 (valid)

*sipaliwini,* *Crenicichla,* Ploeg, 1987 (valid)

*stocki,* *Crenicichla,* Ploeg, 1991 (valid)

*sveni,* *Crenicichla,* Ploeg, 1991 (valid)

*taikyra,* *Crenicichla,* Casciotta, Almirón, Aichino, Gómez, Piálek & Říčan, 2013 (valid)

*tapii,* *Crenicichla,* Piálek, Dragová, Casciotta, Almirón & Říčan, 2015 (valid)

*tendybaguassu,* *Crenicichla,* Lucena & Kullander, 1992 (valid)

*ternetzi,* *Crenicichla,* Norman, 1926 (valid)

*tesay,* *Crenicichla,* Casciotta & Almirón, 2008 (valid)

*tigrina,* *Crenicichla,* Ploeg, Jegu & Ferreira, 1991 (valid)

*tingui,* *Crenicichla,* Kullander & Lucena, 2006 (valid)

*tuca,* *Crenicichla,* Piálek, Dragová, Casciotta, Almirón & O. Říčan, 2015 (valid)

*urosema,* *Crenicichla,* Kullander, 1990 (valid)

*vaillanti,* *Crenicichla,* Pellegrin, 1903 (valid)

*virgatula,* *Crenicichla,* Ploeg, 1991 (valid)

*vittata,* *Crenicichla,* Heckel, 1840 (valid)

*wallacii,* *Crenicichla,* Regan, 1905 (valid)

*yaha,* *Crenicichla,* Casciotta, Almirón & Gómez, 2006 (valid)

*yjhui,* *Crenicichla,* Piálek, Casciotta, Almirón & Říčan, 2019 (valid)

*ypo,* *Crenicichla,* Casciotta, Almirón, Piálek, Gómez & Říčan, 2010 (valid)

*zebrina,* *Crenicichla,* Montaña, López-Hernández & Taphorn, 2008 (valid)

***Crenicichla* species groups:**

Acutirostris

*acutirostris, multispinosa, percna, phaiospilus, ternetzi, tigrina, zebrina,*

Lacustris

*celidochilus, haroldoi, iguapina, iguassuensis, jaguarensis, jupiaensis, lacustris, maculata, mucuryna, niederleinii, punctata, tingui, vittata, gillmorlisi, hu, lucenai, mandelburgeri, taikyra, tapii, tesay, tuca, yaha, yjhui, ypo,*

Lugubris

*adspersa, cincta, dandara*, *johanna, lenticulata, lugubris, marmorata, monicae, rosemariae, strigata,*

Macrophthalma

*macrophthalma,*

Missioneira

*empheres, hadrostigma, igara, jurubi, minuano, missioneira, tendybaguassu,*

Reticulata

*cametana, cyanonotus, cyclostoma, geayi, jegui, reticulata, sedentaria, semifasciata, stocki,*

Saxatilis

*albopunctata, alta, anthurus, brasiliensis, britskii, chicha, coppenamensis, frenata, hemera, hummelincki, inpa, isbrueckeri, labrina, lepidota, lucius, menezesi, nickeriensis, pellegrini, ploegi, proteus, pydanielae, santosi, saxatilis, semicincta, sipaliwini, sveni, vaillanti,*

Scottii

*gaucho, prenda, scottii,*

Wallacii

*anamiri, compressiceps, heckeli, notophthalmus, regani, urosema, virgatula, wallacii,*

***Crenicichla* synonyms: 35**

**Included in present study (in yellow): 10**

*amarus,* *Labrus,* Larrañaga, 1923 (synonym)

*astroblepa,* *Crenicichla,* Ploeg, 1986 (synonym of *cametana*)

*biocellata,* *Crenicichla,* von Ihering, 1914 (synonym)

*biocellatus,* *Sparus,* Walbaum, 1792 (synonym of *saxatilis*)

*brasilliensis fasciata,* *Crenicichla,* Pellegrin, 1904 (synonym)

*cardiostigma,* *Crenicichla,* Ploeg, 1991 (synonym)

*clancularia,* *Crenicichla,* Ploeg, 1991 (synonym)

*dorsocellata,* *Crenicichla,* Haseman, 1911 (synonym)

*edithae,* *Crenicichla,* Ploeg, 1991 (synonym of *lepidota*)

*elegans,* *Crenicichla,* Steindachner, 1881 (synonym)

*fasciata,* *Cychla,* Jardine, 1843 (synonym)

*funebris,* *Crenicichla,* Heckel, 1840 (synonym)

*guentheri,* *Crenicichla,* Ploeg, 1991 (synonym)

*chacoensis,* *Acharnes,* Holmberg, 1891 (synonym)

*johanna carsevennensis,* *Crenicichla,* Pellegrin, 1905 (synonym)

*lacustris semifasciata,* *Crenicichla,* Devincenzi, 1939 (synonym of *scottii*)

*menezesi,* *Crenicichla,* Ploeg, 1991 (synonym)

*multidens,* *Crenicichla* (*Batrachops*), Steindachner , 1915 (synonym)

*nanus,* *Crenicichla,* Regan , 1913 (synonym)

*nemopterus,* *Batrachops,* Fowler , 1940 (synonym)

*nhoquunda*, *Sparus,* Lacepède, 1802 (synonym)

*nijsseni,* *Crenicichla,* Ploeg, 1991 (synonym)

*obtusirostris,* *Crenicichla,* Günther, 1862 (synonym)

*ocellata,* *Boggiania,* Perugia, 1897 (synonym)

*ornata,* *Crenicichla,* Regan, 1905 (synonym)

*pavo*, *Sparus,* Lacepède , 1802 (synonym)

*pavoninus,* *Scarus,* Gronow, 1854 (synonym)

*polysticta*, *Crenicichla,* Hensel , 1870 (synonym)

*proteus argynnis,* *Crenicichla,* Cope, 1872 (synonym)

*pterogramma,* *Crenicichla,* Fowler, 1914 (synonym)

*punctulatus,* *Batrachops,* Regan, 1905 (synonym)

*rufescens,* *Scarus,* Walbaum, 1792 (synonym)

*rutilans,* *Cychla,* Jardine, 1843 (synonym)

*santaremensis,* *Crenicichla,* Haseman , 1911 (synonym)

*simoni,* *Crenicichla,* Haseman, 1911 (synonym)

***Teleocichla*, all described species (9):**

**valid: 9**

**Included in present study (in green): 5**

*Teleocichla centisquama* Zuanon & Sazima, 2002 (valid)

*Teleocichla centrarchus* Kullander, 1988 (valid)

*Teleocichla cinderella* Kullander, 1988 (valid)

*Teleocichla gephyrogramma* Kullander, 1988 (valid)

*Teleocichla monogramma* Kullander, 1988 (valid)

*Teleocichla preta* Varella, Zuanon, Kullander & López-Fernández, 2016 (valid)

*Teleocichla prionogenys* Kullander, 1988 (valid)

*Teleocichla proselytus* Kullander, 1988 (valid)

*Teleocichla wajapi* Varella & Moreira, 2013 (valid)

**Appendix 2 Nominal species of *Crenicichla* and *Teleocichla* (by year of description)**

Described species included (132):

valid: 96 synonym: 36

Till 1800: 4

Till 1900: 33

Till 2000: 72

From 2000: 22

*Sparus saxatilis* Linnaeus, 1758 (valid)

*Perca brasiliensis* Bloch, 1792 (valid)

*Sparus biocellatus* Walbaum, 1792 (synonym)

*Scarus rufescens* Walbaum, 1792 (synonym)

*Sparus nhoquunda* Lacepède, 1802 (synonym)

*Sparus pavo* Lacepède , 1802 (synonym)

*Crenicichla lepidota* Heckel, 1840 (valid)

*Crenicichla lugubris* Heckel, 1840 (valid)

*Batrachops semifasciatus* Heckel, 1840 (valid)

*Crenicichla vittata* Heckel, 1840 (valid)

*Crenicichla macrophthalma* Heckel, 1840 (valid)

*Crenicichla adspersa* Heckel, 1840 (valid)

*Crenicichla johanna* Heckel, 1840 (valid)

*Crenicichla lenticulata* Heckel, 1840 (valid)

*Batrachops reticulatus* Heckel, 1840 (valid)

*Crenicichla funebris* Heckel, 1840 (synonym)

*Cychla fasciata* Jardine, 1843 (synonym)

*Cychla rutilans* Jardine, 1843 (synonym)

*Scarus pavoninus* Gronow, 1854 (synonym)

*Cycla lacustris* Castelnau, 1855 (valid)

*Cycla conibos* Castelnau, 1855 (valid)

*Crenicichla frenata* Gill, 1858 (valid)

*Crenicichla johanna* var. B *strigata* Günther, 1862 (valid)

*Crenicichla acutirostris* Günther, 1862 (valid)

*Crenicichla obtusirostris* Günther, 1862 (synonym)

*Crenicichla cyanonotus* Cope, 1870 (valid)

*Crenicichla punctata* Hensel, 1870 (valid)

*Crenicichla polysticta* Hensel, 1870 (synonym)

*Crenicichla lucius* Cope, 1871 (valid)

*Crenicichla proteus* Cope, 1871 (valid)

*Crenicichla anthurus* Cope, 1871 (valid)

*Crenicichla proteus argynnis* Cope, 1872 (synonym)

*Crenicichla elegans* Steindachner, 1881 (synonym)

*Acharnes niederleinii* Holmberg, 1891 (valid)

*Acharnes chacoensis* Holmberg, 1891 (synonym)

*Crenicichla saxatilis* var. *semicincta* Steindachner, 1892 (valid)

*Boggiania ocellata* Perugia, 1897 (synonym)

*Crenicichla multispinosa* Pellegrin, 1903 (valid)

*Crenicichla geayi* Pellegrin, 1903 (valid)

*Crenicichla vaillanti* Pellegrin, 1903 (valid)

*Crenicichla brasiliensis marmorata* Pellegrin, 1904 (valid)

*Crenicichla saxatilis* var. *albopunctata* Pellegrin, 1904 (valid)

*Crenicichla brasiliensis* var. *fasciata* Pellegrin, 1904 (synonym)

*Crenicichla cincta* Regan, 1905 (valid)

*Crenicichla wallacii* Regan, 1905 (valid)

*Crenicichla ornata* Regan, 1905 (synonym)

*Crenicichla johanna* var. *carsevennensis* Pellegrin, 1905 (synonym)

*Batrachops punctulatus* Regan , 1905 (synonym)

*Batrachops scottii* Eingenmann, 1907 (valid)

*Crenicichla cametana* Steindachner, 1911 (valid)

*Crenicichla dorsocellata* Haseman, 1911 (synonym)

*Crenicichla iguassuensis* Haseman, 1911 (valid)

*Crenicichla jaguarensis* Haseman, 1911 (valid)

*Crenicichla santaremensis* Haseman , 1911 (synonym)

*Crenicichla simoni* Haseman, 1911 (synonym)

*Crenicichla alta* Eigenmann, 1912 (valid)

*Crenicichla notophthalmus* Regan, 1913 (valid)

*Crenicichla nanus* Regan , 1913 (synonym)

*Crenicichla mucuryna* von Ihering, 1914 (valid)

*Crenicichla biocellata* von Ihering, 1914 (synonym)

*Crenicichla pterogramma* Fowler, 1914 (synonym)

*Crenicichla* (*Batrachops*) *multidens* Steindachner , 1915 (synonym)

*Labrus amarus* Larrañaga, 1923 (synonym)

*Crenicichla ternetzi* Norman, 1926 (valid)

*Cychla labrina* Spix & Agassiz, 1931 (valid)

*Crenicichla lacustris* var. *semifasciata* Devincenzi, 1939 (synonym)

*Batrachops nemopterus* Fowler , 1940 (synonym)

*Crenicichla jupiaensis* Britski & Luengo, 1968 (valid)

*Crenicichla haroldoi* Luengo & Britski, 1974 (valid)

*Crenicichla britskii* Kullander, 1982 (valid)

*Crenicichla compressiceps* Ploeg, 1986 (valid)

*Crenicichla cyclostoma* Ploeg, 1986 (valid)

*Crenicichla jegui* Ploeg, 1986 (valid)

*Crenicichla sedentaria* Kullander, 1986 (valid)

*Crenicichla astroblepa* Ploeg, 1986 (synonym)

*Crenicichla celidochilus* Casciotta, 1987 (valid)

*Crenicichla coppenamensis* Ploeg, 1987 (valid)

*Crenicichla nickeriensis* Ploeg, 1987 (valid)

*Crenicichla sipaliwini* Ploeg, 1987 (valid)

*Crenicichla regani* Ploeg, 1989 (valid)

*Crenicichla heckeli* Ploeg, 1989 (valid)

*Crenicichla hemera* Kullander, 1990 (valid)

*Crenicichla urosema* Kullander, 1990 (valid)

*Crenicichla percna* Kullander, 1991 (valid)

*Crenicichla hummelincki* Ploeg, 1991 (valid)

*Crenicichla inpa* Ploeg, 1991 (valid)

*Crenicichla isbrueckeri* Ploeg, 1991 (valid)

*Crenicichla menezesi* Ploeg, 1991 (synonym)

*Crenicichla pellegrini* Ploeg, 1991 (valid)

*Crenicichla phaiospilus* Kullander, 1991 (valid)

*Crenicichla pydanielae* Ploeg, 1991 (valid)

*Crenicichla santosi* Ploeg, 1991 (valid)

*Crenicichla stocki* Ploeg, 1991 (valid)

*Crenicichla sveni* Ploeg, 1991 (valid)

*Crenicichla tigrina* Ploeg, Jegu & Ferreira, 1991 (valid)

*Crenicichla virgatula* Ploeg, 1991 (valid)

*Crenicichla guentheri* Ploeg, 1991 (synonym)

*Crenicichla cardiostigma* Ploeg, 1991 (synonym)

*Crenicichla clancularia* Ploeg, 1991 (synonym)

*Crenicichla edithae* Ploeg, 1991 (synonym)

*Crenicichla nijsseni* Ploeg, 1991 (synonym)

*Crenicichla missioneira* Lucena & Kullander, 1992 (valid)

*Crenicichla gaucho* Lucena & Kullander, 1992 (valid)

*Crenicichla igara* Lucena & Kullander, 1992 (valid)

*Crenicichla jurubi* Lucena & Kullander, 1992 (valid)

*Crenicichla minuano* Lucena & Kullander, 1992 (valid)

*Crenicichla prenda* Lucena & Kullander, 1992 (valid)

*Crenicichla tendybaguassu* Lucena & Kullander, 1992 (valid)

*Crenicichla rosemariae* Kullander, 1997 (valid)

*Crenicichla tingui* Kullander & Lucena, 2006 (valid)

*Crenicichla iguapina* Kullander & Lucena, 2006 (valid)

*Crenicichla maculata* Kullander & Lucena, 2006 (valid)

*Crenicichla yaha* Casciotta, Almirón & Gómez, 2006 (valid)

*Crenicichla hadrostigma* Lucena, 2007 (valid)

*Crenicichla empheres* Lucena, 2007 (valid)

*Crenicichla zebrina* Montaña, López-Hernández & Taphorn, 2008 (valid)

*Crenicichla tesay* Casciotta & Almirón, 2008 (valid)

*Crenicichla mandelburgeri* Kullander, 2009 (valid)

*Crenicichla hu* Piálek, Říčan, Casiotta & Almiron, 2010 (valid)

*Crenicichla ypo* Casciotta, Almirón, Piálek, Gómez & Říčan, 2010 (valid)

*Crenicichla chicha* Varella, Kullander & Lima, 2012 (valid)

*Crenicichla gillmorlisi* Kullander & Lucena, 2013 (valid)

*Crenicichla taikyra* Casciotta, Almirón, Aichino, Gómez, Piálek & Říčan, 2013 (valid)

*Crenicichla lucenai* Mattos, Schindler, Ottoni & Cheffe, 2014 (valid)

*Crenicichla anamiri* Ito & Py-Daniel, 2015 (valid)

*Crenicichla tuca* Piálek, Dragová, Casciotta, Almirón & O. Říčan, 2015 (valid)

*Crenicichla monicae* Kullander & Varella, 2015 (valid)

*Crenicichla tapii* Piálek, Dragová, Casciotta, Almirón & Říčan, 2015 (valid)

*Crenicichla dandara* Varella & Ito, 2018 (valid)

*Crenicichla ploegi* Varella, Loeb, Lima & Kullander, 2018 (valid)

*Crenicichla yjhui* Piálek, Casciotta, Almirón & Říčan, 2019 (valid)

**Appendix 3**

**Putativelly underscribed species in cichlid hobby literature**

***Teleocichla*:**

**Stawikowski and Werner, 2004, potentially undescribed species (11):**

sp. Araguari

sp. Curuá

sp. Jari

sp. Paru

sp. Tapajós I

sp. Tapajós II

sp. Tocantins

sp. Xingu I

sp. Xing II

sp. Xingu III

sp. Xingu IV

***Crenicichla:***

***Cichlidae.com*, potentially undescribed species (39):**

sp. 'acutirostris madeira' (undescribed)

sp. 'approuage' (undescribed)

sp. 'arapiuns' (undescribed)

sp. 'arapiuns lugubris' (undescribed)

sp. 'aripuana' (undescribed)

sp. 'atabapo' (undescribed)

sp. 'belem' (undescribed)

sp. 'belly crawler' (undescribed)

sp. 'british guyana i' (undescribed)

sp. 'british guyana ii' (undescribed)

sp. 'casiquiare' (undescribed)

sp. 'false anthurus' (undescribed)

sp. 'giraff' (undescribed)

sp. 'heckelii cumina' (undescribed)

sp. 'inirida i' (undescribed)

sp. 'inirida ii' (undescribed)

sp. 'inirida iii' (undescribed)

sp. 'jegui' (undescribed)

sp. 'jurua eirunepe' (undescribed)

sp. 'lagunera' (undescribed)

sp. 'lugubris atabapo' (undescribed)

sp. 'madeira' (undescribed)

sp. 'maici-mirim madeira' (undescribed)

sp. 'missioneira rio forquilha' (undescribed)

sp. 'orinoco dwarf' (undescribed)

sp. 'orinoco puerto ayacucho' (undescribed)

sp. 'sao francisco' (undescribed)

sp. 'strigata orinoco' (undescribed)

sp. 'tapajos red' (undescribed)

sp. 'tapajos shoulder spot' (undescribed)

sp. 'tocantins i' (undescribed)

sp. 'tocantins ii' (undescribed)

sp. 'tocantins iii' (undescribed)

sp. 'trombetas' (undescribed)

sp. 'uruguay black line' (undescribed)

sp. 'venezuela' (undescribed)

sp. 'xingu i' (undescribed)

sp. 'xingu ii' (undescribed)

sp. 'xingu III' (undescribed)

**Stawikowski and Werner, 2004, potentially undescribed species (64+10):**

sp. Apui

sp. aff. acutirostris Aripuaná

sp. aff. acutirostris Maici

sp. aff. acutirostris.Nhamundá

sp. aff. gaucho

sp. aff. jegui

sp. aff. lugubris Orinoco

sp. aff. lugubris Sipapo/Atabapo

sp. aff. lugubris Tapajós

sp. aff. lugubris Uaupés

sp. aff. lugubris Xingu (sp. Xingu II)

sp. aff. sedentaria

sp. Altamira

sp. Amapú

sp. Arapiuns

sp. Aripuana

sp. Artigas

sp. Atabapo

sp. Cayenne

sp. cf. Sao Francisco

sp. Cuyuni

sp. Ecuador

sp. Erepecuru

sp. Essequibo

sp. Guaniamo/Guariquito

sp. Gurupi

sp. Guyana

sp. Guyana II

sp. Jabuti

sp. Juma

sp. Juruá

sp. Leopard

sp. lnirida

sp. lnirida I

sp. lnirida II

sp. lnirida III

sp. lnirida IV

sp. ltapemirim

sp. ltapicuru

sp. Maici-Mirim

sp. Malaria

sp. Manaus

sp. Nhamundá

sp. Orinoco

sp. Orituco

sp. Pernambuco

sp. Portel /Pacajá

sp. Puerto Gaitián

sp. Purus

sp. Ribeira

sp. Rio Negro

sp. Ruropolis

sp. Sao Francisco

sp. Sinóp

sp. Suiá-Missu

sp. Tapajós

sp. Tocantins I

sp. Tocantins II

sp. Uaupés

sp. Xingu

sp. Xingu I

sp. Xingu III

sp. Xingu IV

sp. Yuruán

cf. cyanonotus

cf. edithae

cf. inpa

cf. lepidota

cf. lugubris

cf. marmorata

cf. menezesi

cf. microcephala

cf. saxatilis

cf. wallacii
