# Supplementary figures and images for: MtDNA species-level phylogeny and delimitation support significantly underestimated diversity and endemism in the largest Neotropical cichlid genus (Cichlidae: Crenicichla)
Source: PeerJ. 2021 Nov 9;9:e12283. doi: 10.7717/peerj.12283 (PMC8588857; doi:10.7717/peerj.12283)

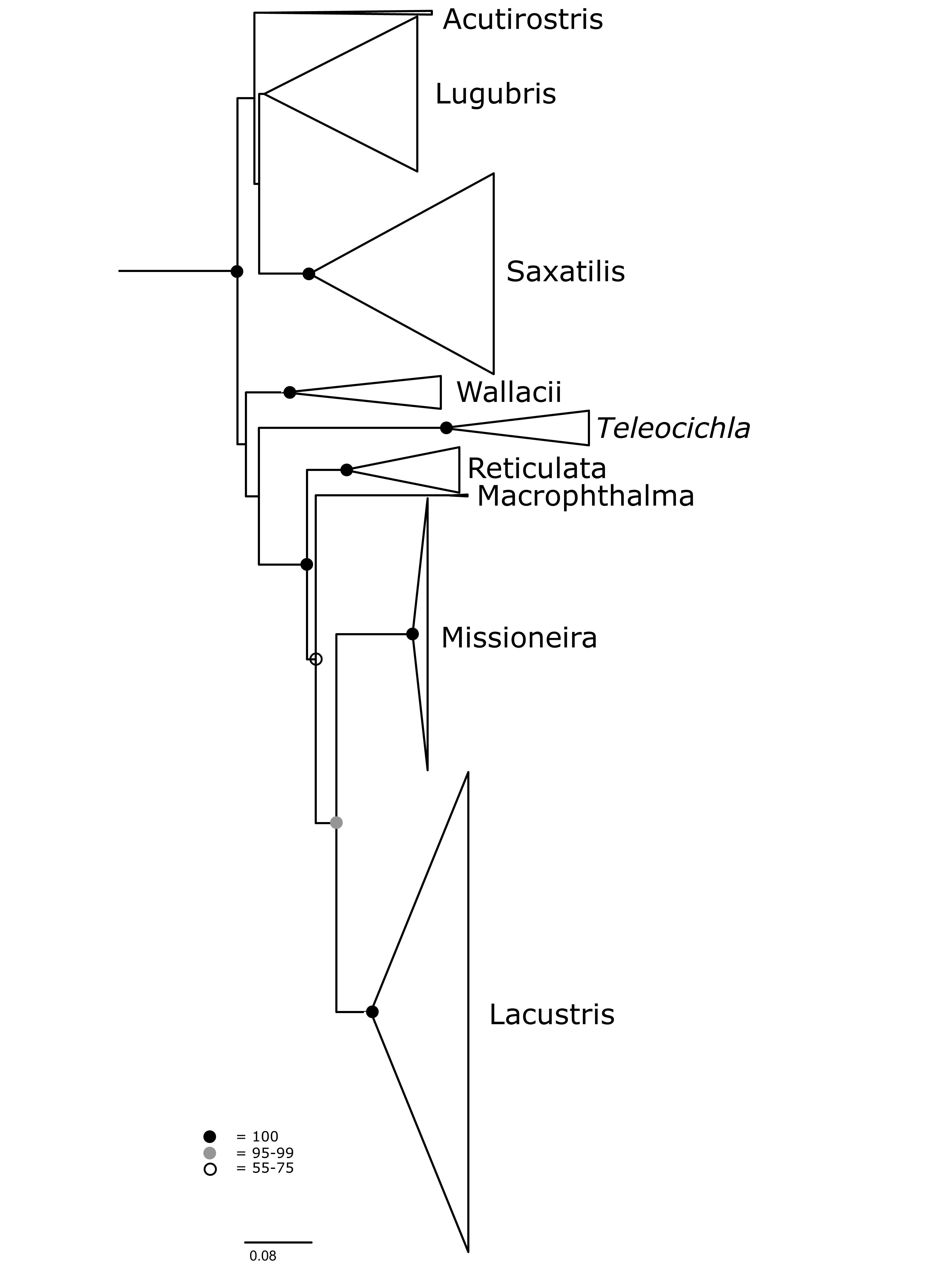

Supplement: Supplemental Information 4 [file peerj-09-12283-s004.png]

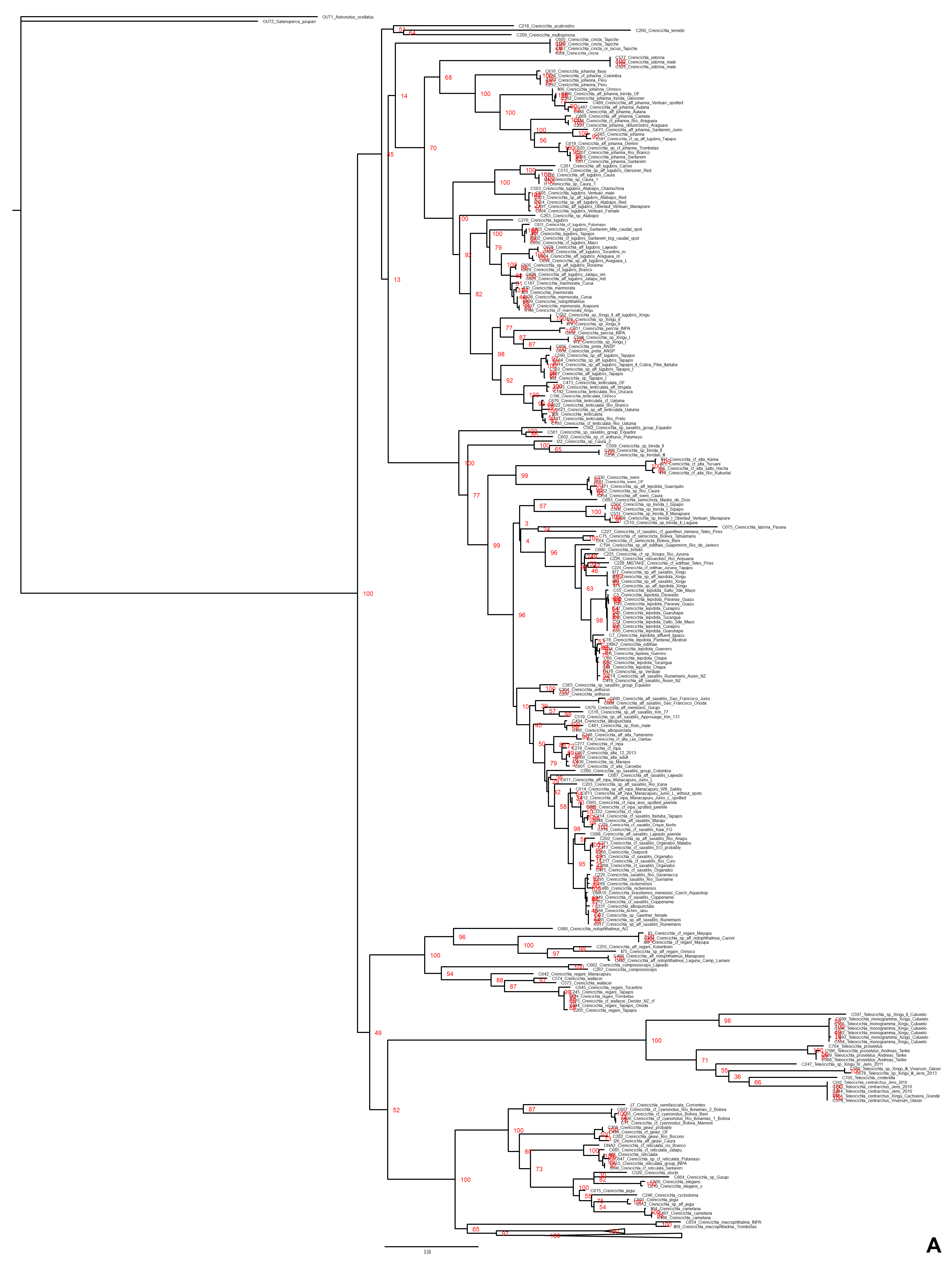

Supplement: Supplemental Information 5 — Showing Teleocichla and the Wallacii, Lugubris, Saxatilis, Reticulata and Macrophthalma groups. [file peerj-09-12283-s005.png]

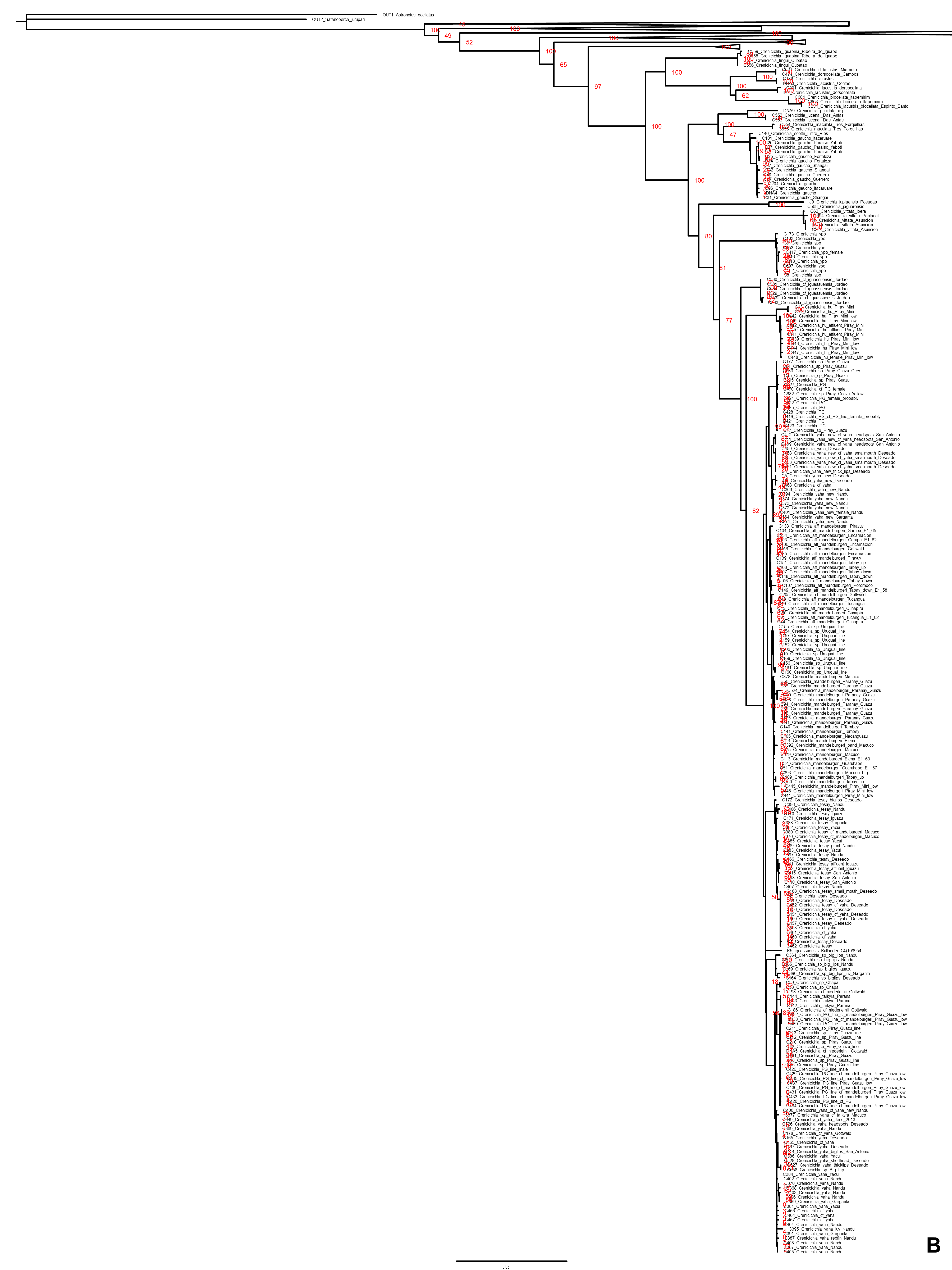

Supplement: Supplemental Information 6 [file peerj-09-12283-s006.png]

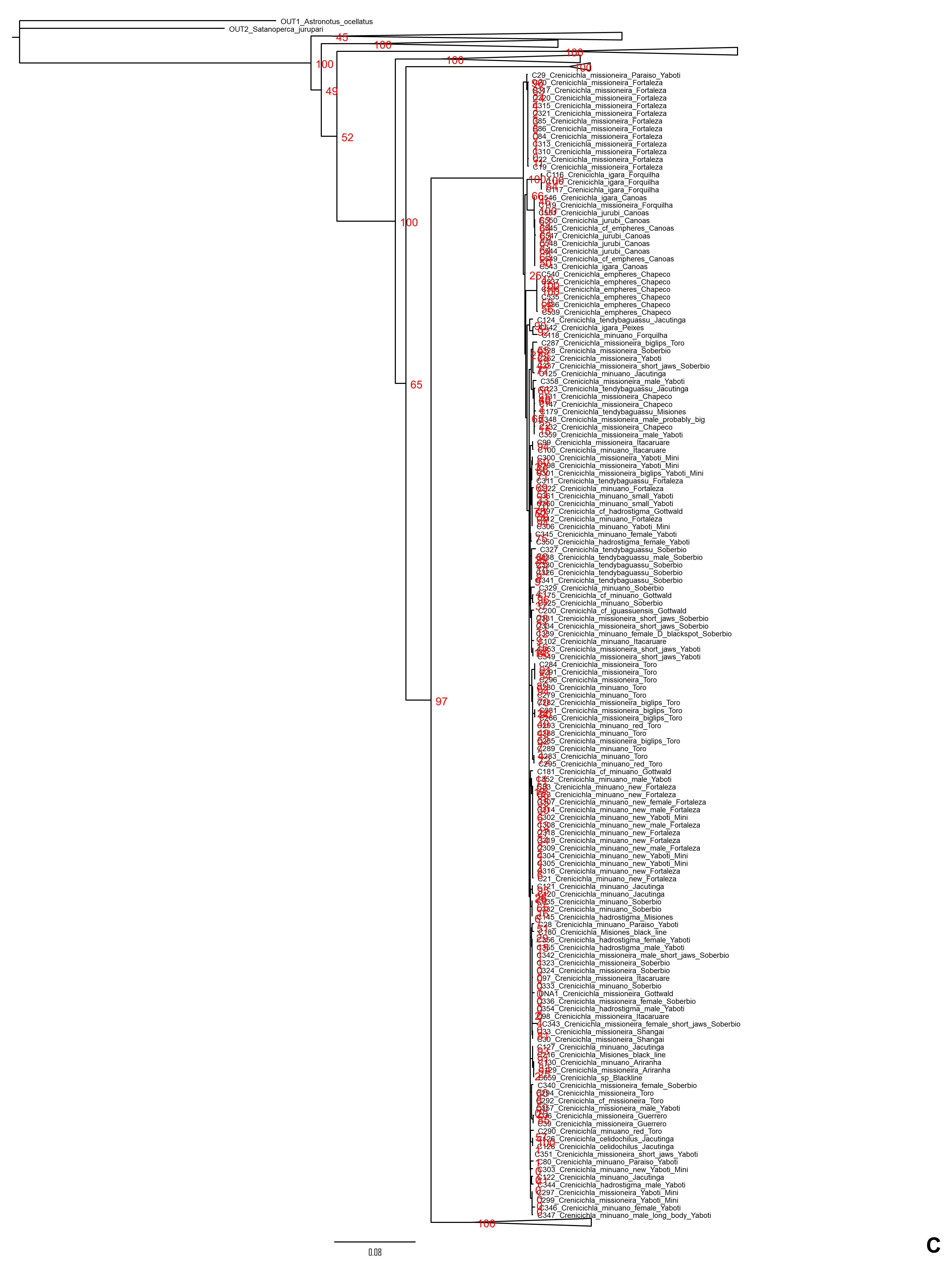

Supplement: Supplemental Information 7 [file peerj-09-12283-s007.png]
